# Replacing paper data collection forms with electronic data entry in the field: findings from a study of community-acquired bloodstream infections in Pemba, Zanzibar

**DOI:** 10.1186/1756-0500-5-113

**Published:** 2012-02-21

**Authors:** Kamala Thriemer, Benedikt Ley, Shaali M Ame, Mahesh K Puri, Ramadhan Hashim, Na Yoon Chang, Luluwa A Salim, R Leon Ochiai, Thomas F Wierzba, John D Clemens, Lorenz von Seidlein, Jaqueline L Deen, Said M Ali, Mohammad Ali

**Affiliations:** 1 International Vaccine Institute, SNU Research Park, San 4-8, Nakseongdae-dong, Gwanak-gu, Seoul, Republic of Korea 151-600; 2 Vienna Biocenter, University of Vienna, Vienna, Austria; 3 Public Health Laboratory-Ivo de Carneri, Pemba, Zanzibar, Tanzania; 4 Menzies School of Health Research, Casuarina, NT, Australia

**Keywords:** Bacteraemia, Handheld computers, Personal digital assistants

## Abstract

**Background:**

Entering data on case report forms and subsequently digitizing them in electronic media is the traditional way to maintain a record keeping system in field studies. Direct data entry using an electronic device avoids this two-step process. It is gaining in popularity and has replaced the paper-based data entry system in many studies. We report our experiences with paper- and PDA-based data collection during a fever surveillance study in Pemba Island, Zanzibar, Tanzania.

**Methods:**

Data were collected on a 14-page case report paper form in the first period of the study. The case report paper forms were then replaced with handheld computers (personal digital assistants or PDAs). The PDAs were used for screening and clinical data collection, including a rapid assessment of patient eligibility, real time errors, and inconsistency checking.

**Results:**

A comparison of paper-based data collection with PDA data collection showed that direct data entry via PDA was faster and 25% cheaper. Data was more accurate (7% versus 1% erroneous data) and omission did not occur with electronic data collection. Delayed data turnaround times and late error detections in the paper-based system which made error corrections difficult were avoided using electronic data collection.

**Conclusions:**

Electronic data collection offers direct data entry at the initial point of contact. It has numerous advantages and has the potential to replace paper-based data collection in the field. The availability of information and communication technologies for direct data transfer has the potential to improve the conduct of public health research in resource-poor settings.

## Background

The case report form (CRF) is a structured questionnaire used to collect data on a participant in research studies. Paper CRFs are commonly used for studies in developing countries but electronic methods of data collection and processing are becoming more popular. Such methods have been considered an efficient way of computerizing information resulting in higher data quality [1-4], and providing a clean and complete database within a shorter period of time [2,3,5]. The use of handheld computers or personal digital assistants (PDAs) has been reported from clinical studies in Gabon [1] and Nicaragua [6]; household surveys in Tanzania [7], Togo, Niger [8], and Burkina Faso [9]; a patient follow-up study (in combination with the use of an electronic medical record system) in Kenya [10]; and a registration and household enumeration that was recently conducted during a mass vaccination campaign in Zanzibar, Tanzania [2].

During the initial phase of a hospital-based fever surveillance study in three district hospitals on Pemba Island, Zanzibar, Tanzania, a number of problems in data collection and entry using paper CRFs were faced. In the light of available reports on PDA use in research from several African countries, including Zanzibar [1,2,7,8,10], PDAs for screening, directing study patient flow, and collection of data were introduced. We report here a comparison of our experiences using paper forms versus PDAs for data collection and management. The objective was to assess the feasibility and acceptability of using electronic data capture for research in a rural and resource-poor setting in sub-Saharan Africa and compare it with paper-based data collection.

## Methods

### Ethics

The study was conducted according to the principles expressed in the Declaration of Helsinki. Written informed consent was obtained from each participant, or from his or her guardian, if the participant was less than 18 years of age at the time of the study. The Zanzibar Research Council Ethics Committee and the Institutional Review Board of the International Vaccine Institute in Seoul, Republic of Korea approved this project.

### Study site

**Figure 1:**
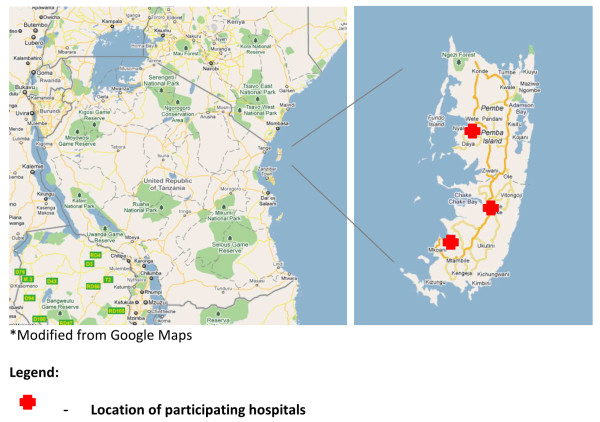
**Study site**.

The study was conducted on Pemba, one of the main islands of the Zanzibar archipelago (Tanzania) located approximately 60 kilometres off the eastern coast of mainland Tanzania (Figure 1). The district hospitals in Chake-Chake, Mkoani, and Wete were centres for participant enrolment into the study. Pemba is a mainly rural area with an approximate population of 500,000 and a total land area of 984 square kilometres [11]. Much of its terrain is hilly, heavily vegetated, and poorly accessible mainly due to unpaved roads. The northern region of Pemba is divided into two districts, Micheweni and Wete, the southern region is divided into Mkoani and Chake-Chake districts. The administrative centre is Chake-Chake, which is located in the district of the same name.

### Study procedures

**Figure 2:**
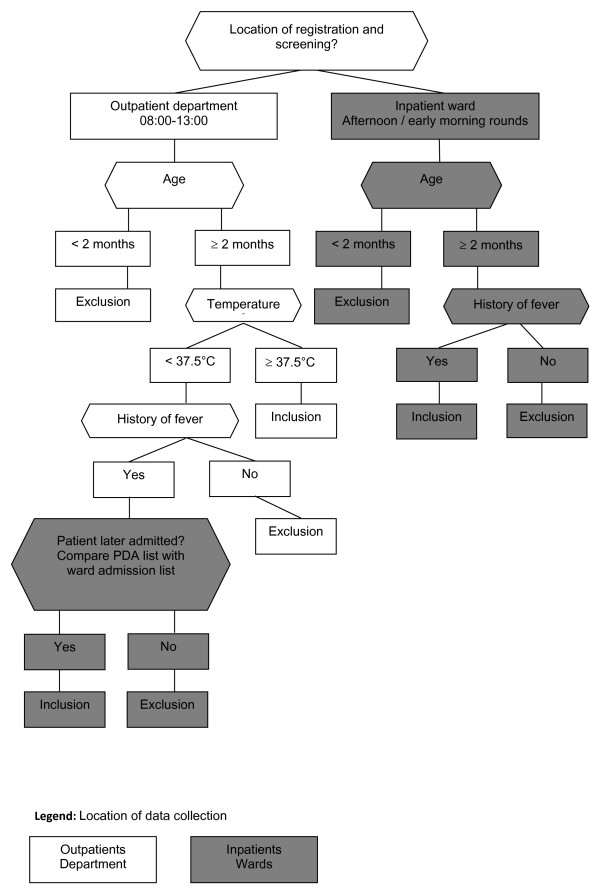
**Flow diagram of the PDA questions used during registration to determine the eligibility of patients presenting to one of the three district hospitals in Pemba, Zanzibar**.

The comparison of the two data collection methods was done as part of a study that assessed the burden of community-acquired bloodstream infections in febrile patients [12]. All patients seeking treatment at one of the three district hospitals were registered and screened for eligibility by study staff upon arrival at the outpatient department (OPD) or the inpatient ward. Inclusion criteria for outpatients were age over 2 months and a recorded temperature of ≥ 37.5°C (axillary); while inclusion criteria for inpatients were any history of fever and age above 2 months. Since registration and screening were performed prior to the decision to admit patients to the ward or to treat as outpatients, current body temperature and history of fever were recorded for all patients. The majority of participants were enrolled in the outpatient department. In the event that a patient did not initially fulfil the inclusion criteria at the time of presentation but was later admitted to the ward, he or she was enrolled into the study as an inpatient. After OPD office hours, patients that were directly admitted were recruited from the inpatient ward. Each enrolled patient was assigned a unique identification (ID) number. The directing, registration, and screening of participants according to the flow of questions appearing in the PDA is shown in Figure 2.

The surveillance was implemented using a stepwise approach, starting in Chake-Chake Hospital with paper-based CRFs in September 2008. Direct data entry using PDAs was later implemented at Chake-Chake Hospital (in March 2009), as well as at Mkoani (in May 2009) and Wete Hospitals (in August 2009), and data collection with PDAs continued at all three locations until December 2010.

### Paper-based case report form and data entry

Data was collected using a 14-page CRF that consisted of four sections: registration, case record (clinical history, physical examination and bedside test results for malaria, glucose and haemoglobin), laboratory results, and outcome. There were a total of 74 paper-based fields to be completed, consisting of 44 multiple choice and 30 open-ended questions. Each CRF was labelled with a consecutive serial number. A unique study ID number was manually assigned to the participant at the time of enrolment. After completion of all four sections, each form was sent to the data management team who manually checked for errors or omissions. Any detected error was referred back to the fieldworker who had completed the respective section of the CRF. This was followed by double data entry of completed forms using Microsoft Access (Microsoft, Seattle, WA, USA), which involved data entry by two different individuals. The two data sets were compared to detect keypunch errors, and any discrepancies were addressed by referring to the source document (CRF). The computerized data were validated by reviewing range and logic errors. Finally, the four sections of the CRF were linked together using the unique ID number.

### PDA-based direct data entry

A total of nine Hewlett Packard (Palo Alto, CA, USA) iPAQ 214 Enterprise Handheld personal digital assistants (PDA) with 4-inch TFT touch screen display and Microsoft Windows Mobile^® ^5.2 operating system were used at the three hospitals. Each PDA has a 2200 mAh lithium ion rechargeable main battery that provides at least six hours of usage. In addition, a backup battery was provided for each operator. Each PDA unit cost approximately USD 340. The PDAs were employed at each of the hospitals five days a week from about 7 am to 4 pm, and the batteries were charged overnight.

The software was developed for the CRF direct data entry using a combination of Visual Studio.Net and Visual Basic.Net (Microsoft, Seattle, WA, USA). To upload and manage the data on a desktop computer, another data management software was developed using Microsoft FoxPro 7.0 (Microsoft, Seattle, WA, USA).

**Figure 3:**
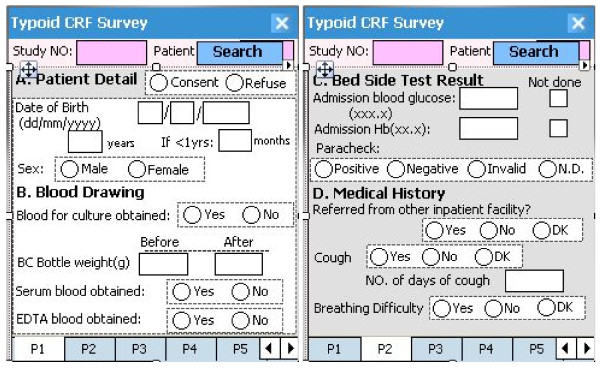
**A sample of case report form screens from the personal digital assistant program**.

The first two sections of the paper-based CRF (registration and case record) were converted into digital versions for direct data entry. Sample screens from the PDA program are shown in Figure 3. The system offered a structured questionnaire to record each patient's information. The data entry fields were restricted to option buttons, check boxes, or fields where appropriate data could be entered. Dropdown menus, skip patterns, and fields requiring data were programmed into the system to prevent errors while navigating through the questionnaire. Once the individual's information was saved, the name and the census ID of the person was shown in the window.

The two remaining sections of the CRF (laboratory results and outcomes) were not replaced with direct data entry methods. These laboratory data were obtained at various times and in locations where PDAs could not always be made available. The paper-based laboratory results and outcome forms were later double-entered into the database as described above.

The data entered in the PDA were collected by a roving field worker on a secure digital memory card, and uploaded into a central data management desktop computer at the end of each day and integrated into the database. The two completed PDA modules and the two paper forms from each patient were linked through the individual ID number in the data management unit. The data were processed in a relational database environment. Further checks such as data integrity and inter-record consistency, which could not be implemented in the PDA system, were completed on a central desktop computer immediately after uploading the data. Queries were sent back to the hospital or laboratory on the following day for resolution. The data entry staff used the edit module in the PDA system to correct erroneous data. The hospital staff could not change data once it had been entered and saved in the PDA.

### Data security and storage

All PDAs and computers were password-protected, and as with completed CRFs, were kept in a safe locker. All data were transferred on a regular basis from the three study hospitals to the central data management unit and uploaded into the central database. The central database was saved with scheduled back-ups.

### Comparison

Both data collection methods were compared regarding training, acceptability, data entry time in minutes per patient file using an average value of 1.4 min/page, data turnaround time in days, omission, accuracy, cost in US Dollar and knowledge transfer. User friendliness/acceptability and ease of implementation was assessed in informal interviews with staff members. Omissions were defined as missing entries. The percentage of omissions were calculated for a subset of 32 variables including age, address, history of fever, weight, temperature, heart rate, blood pressure and clinical signs and symptoms. The accuracy of data was determined by assessing the percentage of typographical errors, decimal point faults and illogical values for the variables mostly affected from this type of errors (glucose, hemoglobin, blood pressure, heart rate and weight of blood culture bottle before and after addition of blood). Accuracy was thereby defined as the absence of typographical errors, decimal point faults, and illogical values.

Cost was calculated using cost for personnel, hardware, printing and database development for paper based data collection and our electronic data collection (including the cost for the 2 parts of the CRF that remained on paper). Frequencies were compared by chi-square test.

## Results

### Problems encountered in using paper-based case report form

The main concern with using the paper forms was the time interval between data collection and data checking prior to computerization. This frequently resulted in the detection of erroneous data at a point when it was too late to make corrections (e.g., after the patient was discharged). The main errors detected were omissions and illogical data.

The inadequate adherence to inclusion and exclusion criteria during the screening process posed an additional challenge. When using paper-based CRFs study enrolment criteria were inconsistently applied, resulting in missing eligible patients and difficulties tracking the proportion of patients missed for the study but were missed, creating a potential bias when reporting the results. These problems led to the introduction of PDAs for electronic data collection.

### Direct data entry using PDAs

The principal challenge in the use of PDAs was the creation of customized software for this study. This task was completed by experienced data managers. This was then followed by two-day training sessions for study personnel before the pilot and full implementation of the PDAs.

#### Screening and registration

The data from all patients screened in the outpatient department (OPD) were entered into PDAs. These included age, body temperature, and history of fever. Based on the screening data, the PDA used an algorithm to determine the eligibility of the patient to be enrolled in the study, and study staff was then alerted to enroll these patients. This algorithm allowed for the different presentation scenarios as described below, and as shown schematically in Figure 2. Firstly, upon registration at the OPD between 8 am to 1 pm, patients above the age of two months and with temperatures of ≥ 37.5°C were found to be eligible and enrolled. Secondly, so as not to miss those who may have had temperatures of < 37.5°C at the OPD but presented with a history of fever and were later admitted to hospital, study staff compared a PDA list of screened patients with the wards' admission lists in the afternoon after OPD closure. If an eligible patient had been admitted to the ward, a pop-up screen would appear to suggest enrolment. Thirdly, study staff registered and screened in the late afternoon and early morning of the following day any patient presenting directly to the ward after OPD office hours. The PDA module assigned a unique ID number to all screened patients, irrespective of patient eligibility. The ID number was used to identify each patient for study purposes and to link his or her case report with laboratory results and outcome.

All eligible patients were handed a one-page paper form at registration upon which the assigned study ID number was indicated. This paper "registration form" represented the only research-related paper document that remained with the patient during his or her entire hospital stay and was than returned to study staff. Apart from providing the patient's ID number to the study nurse, this paper form contained the results of the bedside tests for malaria, glucose and hemoglobin for the clinical management of the patient by the attending clinician and was also used for recording the patient's outcome.

#### Enrolment

Eligible patients were enrolled by a study nurse using a second PDA. The study nurse entered the patient's ID number from the paper "registration form" to bring up the case record module. The PDA-based questionnaire was displayed on several pages. The system would allow continuation to the next page only if all questions had been answered to avoid data omission. Queries that logically depend on a primary question would only pop-up if the answer to the primary questions was positive (e.g., primary question: "Do you have diarrhoea?" If answered with a "Yes", the next question would be "How many days?" If answered with a "No", the module would jump to the next group of related questions). The software included skip patterns so that queries would pop-up only if the participant was of the relevant age or gender. Real-time error checks were included to make sure that illogical data could not be entered. Once the case record form was completed, the file was saved automatically.

Laboratory results continued to be recorded on paper and later double-entered into the database. Similarly, information regarding the patient's outcome (discharged, improved, transferred/referred out, absconded, or died) was recorded on the "registration form" that accompanied the patient during his or her hospital stay. This form was kept on paper since it remained with the patient during his or her hospital stay. This form was returned to study staff upon discharge.

### Comparison of the two data collection methods

A total of 180 patients were enrolled using paper-based data collection, and 2,209 patients were registered and enrolled using PDAs. The use of paper CRFs was compared with direct data entry using PDAs in regards to implementation, outcome, and costs (Table 1)... Most of the staff members had no prior experience in the use of computers and had to familiarise themselves with the device. The duration of the required training on the use of PDAs was initially longer than for paper, but no retraining was required compared with paper forms (Table 1).

**Table 1:** A comparison of paper case report forms with direct data entry using personal digital assistants

	Paper forms	Direct data entry using personal digital assistants
**Training**	Training on error correction, what type of pen to use, etc. according to GCP (takes approx. 1 day with frequent retraining).	Training on use of software and hardware (takes approx. 2 days; no retraining needed).

**User friendliness/acceptability and ease of implementation**	A known method for most staff, and therefore, high acceptability and easy implementation.	Unknown method for staff; high acceptability after training and initial usage; initial implementation requires supervision.

**(Remaining) Data entry time** (paper-> database)*	Double data entry (14 pages): 10 minutes per patient	Double data entry (7 pages): 5 minutes per patient

**Data turnaround time**	5-7 days	Less than 24 hours**

**Data omissions**	Dependent on degree of education and training; high omission (6%) seen in staff not experienced with research.	None since data can't be saved if not all of the questions are answered.**

**Data accuracy**	Dependent on degree of education and training; low accuracy seen in staff not experienced with research (7% non accurate data).	High accuracy due to real-time error and consistency checks (1% non accurate data).**

**Costs**	a) personnel: 2 data entry staff (19 months): USD 5,700 b) hardware: 2 computers (5,000 USD), 1 printer (1,000 USD), filing cabinets (10 × 350 USD), additional space (500 USD): USD 10,000 c) Paper forms: printing CRFs (14 pages × 2,500): USD 2,800 d) IT: database development: USD 5,000 TOTAL: USD 23,500	a) personnel: 1 data entry staff (19 months): USD 2,800 b) hardware: 9 PDAs (each 340 USD), 1 central computer (2,500 USD), 1 printer (1,000 USD), filing cabinets for the remaining paper forms (5 × 350): USD 1,750: USD 8,310 c) Remaining Paper forms: printing lab forms (7pages × 2,500) and outcome forms(1 page × 2,500): USD 1,600 d) IT: database development USD 5,000: TOTAL: USD 17,710

**Transferability to next project**	Knowledge can be transferred to the next project. Hardware can be recycled for the next study.	Knowledge can be transferred to the next project. PDAs and other hardware can be recycled for the next study.

*using an average value of 1,4 min/page as used for staff requirement planning. **excluding the part that remained on paper instead of excluding data turnaround time for the parts that remained on paper

We found that data collection using PDAs was more accurate and complete than paper-based data collection. The rate of omitted information among 32 variables collected in the hospital was 6% (342/5760; 95%CI: 5.4 to 6.6) for the paper based-data collection compared to none in the electronic data collection (*p *< 0.05). Mistakes such as typographical errors, decimal point faults or illogical values for the variables glucose, hemoglobin, blood pressure, heart rate and culture bottle weight before and after incubation of blood were reduced from 7% (65/900; 95%CI: 5.7 to 9.1) in the paper-based questionnaires to 1% (95/11,045; 95%CI: 0.7 to 1.0) in the electronic data collection (*p *< 0.05) respectively (Table 2).

**Table 2:** Omissions and accuracy for paper-based versus PDA based data collection

	Paper			PDA			p
	**Number of variables checked**	**Number of records**	**Number (%) of omissions**	**Number of variables checked**	**Number of records**	**Number (%) of omissions**	

**Omissions**	32	180	342 (6%)	5	2209	0 (0%)	< 0.05

**Accuracy**	5	180	65 (7%)	5	2209	95 (1%)	< 0.05

For a 19-month study period, a total expenditure of USD 23,500 was calculated for the paper-based data collection and entry, compared with USD 17,710 for the PDA-based system (Table 1). In addition, PDAs can be re-used in a subsequent study.

## Discussion

Electronic data collection in this rural setting in sub-Saharan Africa using handheld devices was found to be superior to that of a paper-based system in regards to accuracy and completeness of data. Although the capital costs for the initial setup of the PDA-based data entry was higher than that for the paper-based data entry method, this was offset by the lower number of data entry clerks, computers, and printing required. This resulted in savings of approximately 25% of the cost needed for a paper-based data collection. Furthermore, PDAs can be re-used in subsequent projects, further reducing the costs for data collection. Using electronic devices for registration and screening of patients prompted study staff to include eligible participants into the study. The electronic system facilitated the tracking of eligible patients. Finally, the PDAs proved to be popular with the field staff. After an initial training period, none of the users was interested in returning to paper-based data entry.

As described earlier by Ali et al. [2], acceptability of PDA use was high among staff not familiar with computers or PDAs. With increasing use of mobile phones and other similar technologies, operating PDAs, downloading data, and recharging batteries are becoming increasingly familiar concepts. Training on PDA usage did not require substantially more time than training on paper-based data collection. However, creating the software and installing the software at the beginning of the study requires a skilled data manager. While these data management skills are not universally available the skills to manipulate devices such as PDAs are highly marketable and are spreading rapidly.

Similar experiences in regards to completeness of data and turn-around time of available data have been described previously [7] from household surveys and other studies [2]. Discrepancies between paper-based entry and electronic data entry have been quantified by Missouni et al. [1], and were found to be only 1.7% in a clinical study in Gabon. However, data collectors in these studies were well-qualified clinicians, in contrast to this study setting where data were collected by study nurses without any prior research experience. Our experience showed that, especially for study staff with limited or no prior experience with data collection for research studies, electronic data entry enhances accuracy.

The main challenge encountered was difficulty in linking the electronic data with the paper-based laboratory data. Typographical errors occurred when the study ID was recorded from the PDA used for screening and registration on the one-page paper form, and recorded back on the second PDA for enrolment and on the laboratory forms. The linking problem could only be resolved by excluding those unlinked data from the analyses. In the future, this could be avoided by using barcodes.

## Conclusions

Other challenges must be taken into account in future studies. For example, a portable database in each PDA would allow staff to identify returning patients who have been previously enrolled. This has already shown to be successful in other studies [2]. Future use of barcodes or smart phones that are connected via a network to a main server will further enhance accuracy and speed of electronic data entry. Aviles et al. [6] have shown how an information and communication technology system can be used in research settings where data are collected at different points in time and location, using a combination of PDA with wireless data uploaded to a main server. PDAs that are embedded in a GPS system could also be used for supervisory and quality control issues by tracking time, duration, and location of data collection in the field. Continuous synchronization between the mobile devices and the central computer has been used in other sites to improve the performance of direct data entry [6]. We found that data synchronization between a mobile devise and the central computer would have been very time consuming in our rural, remote setting. Thus, in this setting, we preferred to use the mobile devise to collect data, store and copy the data from the SD card to the central computer. In summary, we showed the feasibility and advantages of using electronic data capture in a rural and resource-poor setting in sub-Saharan Africa. Our findings support the growing literature in this field and making electronic data capture increasingly popular.

## Competing interests

The authors declare that they have no competing interests.

## Authors' contributions

KT coordinated the study, was involved in clinical care of patients, and wrote the manuscript, BL coordinated the laboratory work and contributed to the manuscript, SMA supervised the laboratory work, MKP supervised data management, RH contributed to the design of the database, supervised PDA usages and performed PDA training, NYC designed the database, LAS performed day to day data management, RLO provided scientific support to the manuscript, TFW provided scientific support to the manuscript, JDC provided scientific support to the manuscript, LvS provided scientific support to the study and the manuscript, JLD provided scientific support to the study and the manuscript, SMA provided scientific support to the study and the manuscript, MA provided support to the data management team and provided scientific support for the manuscript. All authors have read and approved the final manuscript.
